# A long-term obesogenic high-fat diet in mice partially dampens the anti-frailty benefits of late-life intermittent fasting

**DOI:** 10.1007/s11357-022-00678-1

**Published:** 2022-10-26

**Authors:** Yoko O. Henderson, Nazmin Bithi, Jie Yang, Christopher Link, Aili Zhang, Benjamin Baron, Eran Maina, Christopher Hine

**Affiliations:** 1 Department of Cardiovascular and Metabolic Sciences, Cleveland Clinic Lerner Research Institute, Cleveland, OH 44195 USA; 2 Present address: Department of Pathology, ARUP/University of Utah School of Medicine, Salt Lake City, UT 84112 USA

**Keywords:** High-fat diet (HFD), Every other day fasting (EOD), Aging, Metabolism, Intermittent fasting, Obesity

## Abstract

The global obesity pandemic coupled with ever-growing life expectancies equates to hundreds of millions of individuals with potentially longer but not healthier lives. Aging is one of the risk factors for numerous maladies such as metabolic disorder and frailty, which are exacerbated under obesity. Thus, therapeutic approaches that address obesity to ultimately improve affected individuals’ quality of life and extend their lifespan are needed. We previously reported that the every other day (EOD) fasting initiated late-life improved metabolic, musculoskeletal, and cognitive endpoints in standard rodent diet-fed mice. In the present study, using the same dietary intervention methodology, we tested if 2.5 months of EOD fasting could improve metabolic, physiological, and cognitive endpoints in mice after an 18 month obesogenic high-fat diet (HFD). The positive effects of EOD fasting were generally consistent across the endpoints; EOD fasting decreased total body mass, maintained more %lean mass, improved glucose tolerance and utilization, and improved neuromuscular function. In contrast to our previous study, grip strength, hippocampal-dependent memory, and renal hydrogen sulfide (H_2_S) production were not improved by the HFD EOD fasting. Thus, efficacy for late-life initiated intermittent fasting to improve specific frailty markers may be partially dependent on nutritional compositions of the diet.

**Supplementary Information:**

The online version contains supplementary material available at 10.1007/s11357-022-00678-1.

## Introduction

Obesity is defined as the excessive accumulation of fat in subcutaneous and visceral adipose tissue depots as well as the ectopic lipid buildup in organs such as the liver [1]. The absolute threshold for which this fat accumulation increases an individual’s body mass index (BMI) for obesity diagnosis is debated due to factors such as age, ethnicity, lean mass contributions, and disease state [2]. However, a BMI above 30 kg/m^2^ is usually defined as obese and a risk factor for poor health outcomes in adults [3, 4]. Due to the excessive fat accumulation under obesity, multiple organ systems are negatively impacted, resulting in localized as well as systemic health declines, such as cardiovascular disease, metabolic disorder, diabetes, kidney failure, neurodegeneration, arthritis, inflammation, and severe COVID infection [4, 5].

Despite extensive medical, financial, and public health resources invested, obesity has and continues to be labeled a global pandemic, with over 700 million children and adults falling under this diagnosis [4]. Thus, identifying obesity causes and safe, effective, and lasting interventions to counter it is of tremendous significance.

A growing area of concern regarding obesity is its reciprocal relationship with aging. Aging is a risk factor for obesity due to declines in metabolic fitness and insulin signaling, while obesity itself accelerates the aging process and increases the severity of geriatric syndromes [6–8]. With the global population over age 65 expected to double by the year 2050 [9], and aging itself being a significant risk factor for metabolic syndrome, cardiovascular diseases [10], and neurocognitive deterioration [11], the prevention of obesity- and aging-related health declines are paramount. Thus, as the medical and public health innovations of the last 100 years have yielded longer lifespans primarily due to antibiotics, vaccines, sanitation control, chemotherapeutics, and anti-hypertensive/atherosclerotic drugs, they have not guaranteed improved quality of life at later ages.

Dietary restriction (DR), which includes the subtypes caloric restriction (CR), intermittent fasting, and macronutrient (protein/amino acid) restriction, is a well-established intervention to improve metabolic fitness, decrease adiposity, and slow the aging process [12–22]. We recently reported that late-life initiation of an every other day (EOD) fasting diet for 2.5 months in ~ 20-month-old C57BL/6 mice (estimated equivalent of 55–60 human years [23]) improved multiple components of frailty including body mass composition, glucose metabolism, neuromuscular performance, and hippocampal-dependent memory [12]. These improvements were observed in mice on a standard “healthy” rodent chow composed of 24% kcal from protein, 58% kcal from carbohydrate, and 18% kcal from fat, and primarily only in males but not females. Thus, we questioned if this same late-life initiated EOD fasting regimen could also provide similar benefits in aged male mice when kept on an obesogenic high-fat diet (HFD).

**Fig. 1:**
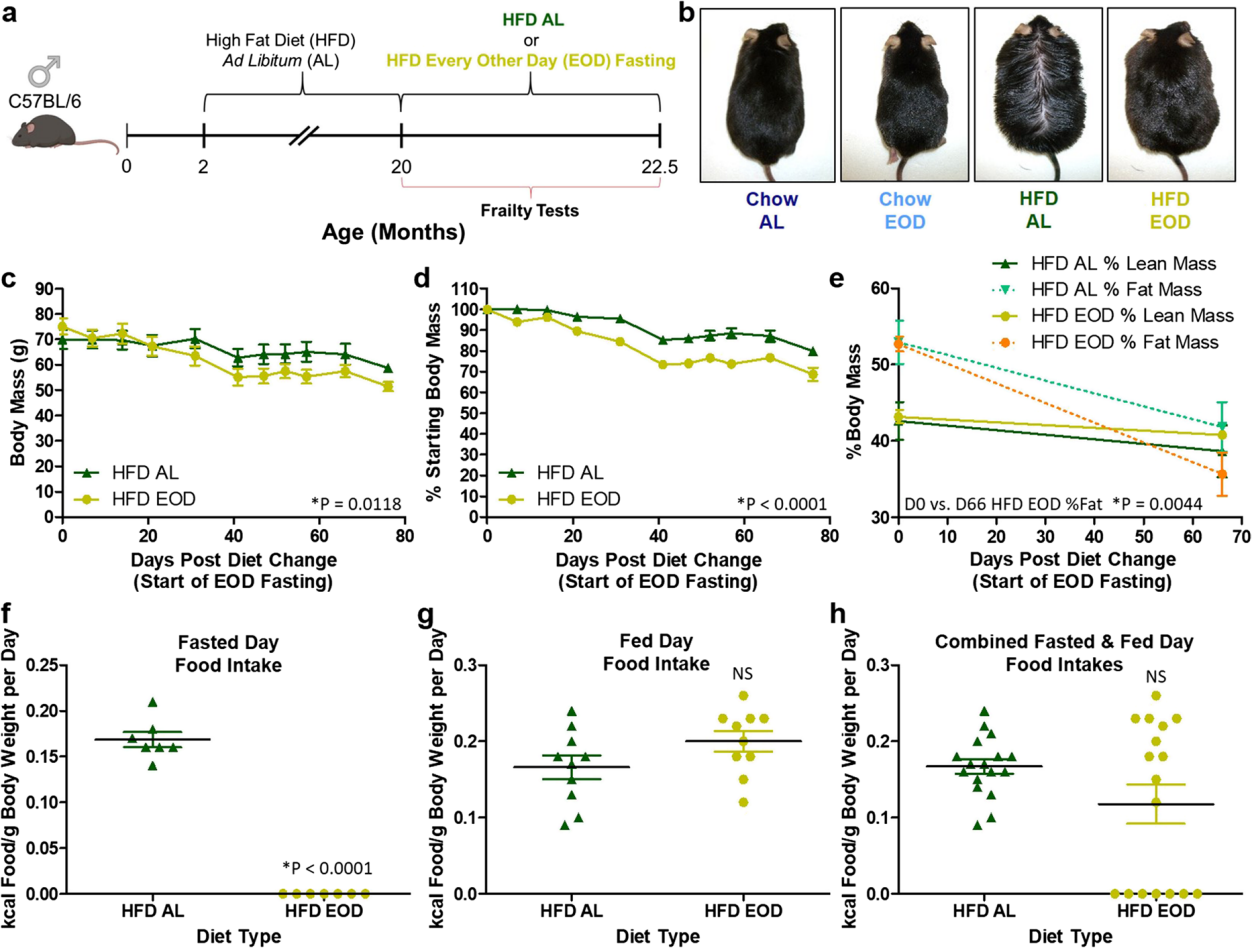
EOD fasting in aged mice on a HFD background promotes overall weight loss and predominantly preserves lean mass. **a** Logistical overview of the experimental timeline. Male C57BL/6 mice were placed on HFD AL at approximately 2 months of age and continued on this diet until 20 months of age. Then, the mice were randomly assigned to the HFD AL group or the HFD EOD group in which the HFD food was provided for 24 h and taken away for 24 h, and this cycle continued for 2.5 months. Frailty was determined using physiological, metabolic, and behavioral tests. **b** Representative images of 22.5-month-old mice under standard chow AL, standard chow EOD, HFD AL, and HFD EOD diets to show reversal of the oily/greasy coat condition in the HFD EOD mice. **c**, **d** Absolute body mass (**c**) and %starting body mass (**d**) in HFD AL (*N* = 2–3) and HFD EOD (*N* = 3) mice throughout the 2.5-month intervention period. **e** Body mass composition (fat and lean masses) normalized to absolute body weight on day 0 and day 66 of the intervention. **f**–**h** Food intake (kcal/g of body weight/day) during the fast days (7 days measured; **f**), fed days (10 days measured; **g**), and combined fast and fed days (17 total days measured; **h**) as calculated on a per cage basis. The figures (**c**–**h**) depict the mean with error bars (± SEM). The asterisks and provided *p* value indicate a significant difference between HFD AL and HFD EOD groups except in **e**, which represents the significance of the difference in the HFD EOD between day 0 and day 66. *N* = 2–3 mice for the HFD AL group and *N* = 3 mice for the HFD EOD group. The reason for only 2 mice in the HFD AL group at the end is due to mortality during the 2.5-month testing period. See also Supplemental Fig. 1

To answer this question, we performed experiments in parallel to the ones recently reported in Henderson et al. [12], except the cohort of male mice utilized here had been kept on an HFD containing 60% kcal from fat since ~ 2 months of age. At ~ 20 months of age, the mice were divided into an HFD ad libitum (AL) control group or an HFD EOD fasting group for 2.5 months with physical, metabolic, and cognitive components of frailty measured throughout the dietary intervention period (Fig. 1a). While we did find improvements in body mass, body mass composition, carbohydrate metabolism, and neuromuscular coordination under the HFD EOD fasting compared to HFD AL, the outcomes were numerically less favorable to HFD EOD fasting than our previous findings under the standard diet fasting. Furthermore, no effects of the EOD fasting were observed on cognitive functions nor on renal H_2_S production in these obese mice. Thus, the impact of the EOD fasting regimen in late-life likely depends on the nutritional composition of the background diet for specific frailty component improvements.

## Materials and methods

As the current study utilizing an HFD was run in parallel with our previous study utilizing standard rodent chow diet [12], detailed methodologies of many of the materials and methods can be found in Henderson et al. [12]. Below, we provide brief materials and methods as well as detail aspects specific to the current study.

### Animals and dietary interventions

All animal experiments adhered to the National Institutes of Health Guide for the Care and Use of Laboratory Animals and were performed with approval from the Cleveland Clinic Institutional Animal Care and Use Committee (IACUC), protocol number 2016–1778 and 2019–2258. Male C57BL/6 mice were obtained from Jackson Laboratories (Stock No. 000664, Jackson Laboratories) and group-housed (3–4 mice per cage) in the Cleveland Clinic Lerner Research Institute Biological Resource Unit on a 14-h light/10-h dark cycle, with temperature between 20 and 23 °C and 30–70% relative humidity. Mice had AL access to standard rodent chow (24% kcal from protein, 58% kcal from carbohydrate, and 18% kcal from fat; Teklad Global Rodent Diet #2918, Envigo) until approximately 2 months of age, and then switched to an obesogenic HFD for 18 months (14% kcal from protein, 26% kcal from carbohydrate, and 60% kcal from fat; Bio-Serve Mouse Diet, High Fat, Soft Pellets, Fisher Scientific #14–726-603). At approximately 20 months of age, the two HFD cages now housing 3 mice per cage (due to the mortal loss of one mouse prior to the intervention start) were randomly assigned to either the HFD EOD fasting intervention or continued on HFD AL access. The EOD fasting regimen was followed as previously reported [12]. Briefly, the EOD fasting regimen consisted of repeated cycles of 24-h consecutive removal of food access with water available AL (fast day) followed by 24-h access to food and water AL (fed day). To prevent disturbances in circadian feeding rhythms between the EOD and AL groups [24, 25], the HFD was provided to or removed from the EOD group 2–3 h before the dark cycle onset (~ 19:00 h). During the course of the study, there was mortality; thus for several of the tests performed, the HFD AL group had *N* = 2 mice, while the HFD EOD group continued to have *N* = 3 mice. Due to this, in some tests in which the before diet intervention measurement was taken to compare to the endpoint measurement, the average value of the two remaining HFD AL animals was used as the endpoint value to compare to the intervention start values for the deceased HFD AL mouse.

### Physiological and metabolic parameters

Body weight was measured throughout the study. Body composition was measured at baseline (day 0) and near the conclusion of the study (day 66 post-diet intervention, aka post-diet change [PDC]) using EchoMRI body composition analysis (EchoMRI, Houston, TX). Glucose tolerance test (GTT) was performed at 13:00 h on day 70 (day 69: fast day) after a 4-h morning fast. The mice were given an intraperitoneal (IP) glucose injection (2 g of glucose/kg body weight), and blood glucose measurements were recorded up to 120 min post-injection (Accu-Chek glucose meter, Roche Diabetes Care, Inc., Indianapolis, IN). The GTT was repeated on day 71 (day 70 fed day).

Whole body metabolic rhythms and parameters were measured by Oxymax-CLAMS (Comprehensive Lab Animal Monitoring System) indirect calorimetry (Columbus Instruments, Columbus, OH) [26] at baseline and at the mid-point of the DI. Double-plotted traces of these measurements were made using the averaged data for each time point within a diet group separated by the fed and fast days with a time of the day expressed in Zeitgeber time (ZT), with most plots containing ~ 220 data points. In these metabolic cages, the HFD EOD mice were fed or fasted at approximately ZT9 daily.

Hydrogen sulfide (H_2_S) production capacities of the liver, kidney, and muscle (quadriceps) were measured by the lead sulfide method as previously described [12, 27, 28]. Briefly, tissues were immediately obtained from euthanized animals (6-month-old AL chow fed and 24-month-old AL chow and AL and EOD HFD fed C57BL/6 mice) and flash-frozen via placement in 1.5-mL tubes and submersion in liquid nitrogen. Flash-frozen tissues were then homogenized and lysed in 1 × passive lysis buffer (Promega, Madison, WI) with the liver and kidney homogenized using a hand held homogenizer (Fisherbrand) and muscle homogenized using a Bullet Blender 24 with Pink Kit lysis beads (Next Advance). One hundred micrograms of normalized protein with 150 µL of reaction mixture containing 10 mM L-cysteine (Cat. #. 168,149, Sigma-Aldrich, St. Louis, MO) and 1 mM pyridoxal phosphate (Cat. # 9255, Sigma-Aldrich, St. Louis, MO) in phosphate-buffered saline were placed in 96-well plates. A 20-mM lead acetate-embedded H_2_S detection filter paper was placed on top of the well plates and incubated at 37 °C for 1–24 h. H_2_S production capacity was quantified by measuring the lead sulfide darkening of the paper using the IntDen function in the ImageJ software package (Rasband, W.S., ImageJ, U.S. National Institutes of Health, Bethesda, MD, USA, https://imagej.nih.gov/ij/, 1997–2018) and subtracting the background values from the reaction mixture blanks.

### Neuromuscular and cognitive/behavioral tasks

The behavioral tasks were conducted in the order of (1) Y maze forced alternation task, (2) forelimb grip strength, (3) open field, (4) novel object location task, and (5) rotarod, and the order was kept constant for each animal. Behavioral sessions were digitally recorded using a CCTV camera mounted to the ceiling positioned directly above the behavioral apparatuses. All recorded behavioral sessions were analyzed using the behavior analysis software TopScanLite Version 2.0 (Clever Sys Inc., Reston, VA).

#### Y maze forced alternation task

Short-term memory was tested using the Y maze forced alternation task at baseline and ~ day 50 post-DI. Mice were trained in the Y maze (each arm 12 cm *H*, 38.5 cm *D*, 9 cm W) surrounded by intra- and extra-maze visual cues. The acquisition trial was 15 min, and the mice did not have access to target arm. After a 2-h inter-trial interval (ITI), a 4-min memory (retention) test was given, and all of the Y maze arms were accessible. The duration and number of entries to each arm and velocity/speed during the retention trial were analyzed.

#### Open field

During the habituation phase of the novel object location test, the mice were given a 10-min open field trial at baseline and ~ day 59 post-DI. An open field (40.5 cm *H* × 51.0 cm *D* × 61.0 cm W) was virtually divided into 3 arenas: outer, middle, and center. Movement and exploratory behavior was digitally recorded for analysis.

#### Novel object location test

Twenty-four hours after the open field test, object location memory was tested using the novel object location test at baseline and ~ day 60 post-DI. In this task, two identical objects were placed at fixed and equidistant locations in the open field. The acquisition trial was 5 min. After a 24-h ITI, a 2-min memory (retention) test was given. Mice were reintroduced to the identical environment and objects, except one of the objects was moved to a new “novel” location, located at the opposite corner relative to its original location. The other object remained at the same location.

#### Muscular strength and neuromuscular function parameters

Forelimb grip strength was assessed at baseline and ~ day 48 using a grip strength meter (Columbus Instruments, Columbus, OH). Each mouse was given five trials, and average grip strength adjusted to total body mass and lean mass was recorded. Motor coordination was tested using a rotarod (Columbus Instruments, Columbus, OH). In this test, the mice were given an acclimation trial (4 rpm for 30 s), followed by a total of four trials (max duration: 300 s, max speed: 50 rpm, acceleration: 1 rpm inclement per 11 s per trial) with 5-min ITI. Latency and speed at which the mouse fell from or passively rotated on the revolving rod were recorded in trials 1–4 as a measure of motor coordination.

### Statistical analyses

Figures depict the means ± SEM with *N* of 2–3 mice per group, with technical and/or repeated measurements as indicated in the figure legends and/or this “Statistical analyses” section. The behavior analysis software TopScanLite Version 2.0 (Clever Sys Inc., Reston, VA) was used for quantifying the behavioral tasks. All data were analyzed using Excel (Microsoft) and GraphPad Prism for Windows (GraphPad Software, Inc.). Results were considered statistically significant when *p* values were less than 0.05, with *p* values provided in the figures and/or figure legends. Source data and uncropped images used to generate the figures and their related statistics and tables are included as a supplemental GraphPad Prism file.

Body mass and change in body mass analyses between HFD AL vs. HFD EOD were performed as paired *t*-tests, with pairing done for each respective day measurements were taken. Comparison of before diet change to after diet change weights and body mass composition was done by unpaired Student’s *t*-test. For manual food intake analysis, unpaired *t*-tests were performed between HFD AL vs. HFD EOD on fasted days (a total of 7 different days measured), fed days (a total of 10 different days measured), and combined fasted and fed days (a grand total of 17 different days food intake was measured on a cage by cage basis). For automatic feeding analysis from the metabolic chambers, paired *t*-tests were performed for each animal’s time point reading (a total of ~ 220 plotted/animal) to account for circadian differences in feeding patterns throughout the day. Likewise, RER, vCO_2_, vO_2_, heat, horizontal beam crossings, and rearing data obtained from the metabolic chambers were also analyzed by paired *t*-tests between HFD AL versus HFD EOD, with pairings for each animal’s time point reading (~ 220 time points plotted/animal). This pairing enables normalization for the impact circadian rhythm has on these parameters at specific time points throughout the day. This is similar to performing area under the curve (AUC) analysis, but helps further take into account the expected circadian shifts. GTT analysis was performed as a paired *t*-test between HFD AL versus HFD EOD, with pairing done for each animal’s blood glucose value at each of the 5 time points during the 120-min GTT. Grip strength was analyzed via 2-way ANOVA. Post-diet change (PDC) rotarod performance between HFD AL versus HFD EOD was analyzed as a paired *t*-test, with pairings done for each animal’s duration and speed values at each of the 4 individual time trials. For cognitive tests such as Y maze, open field, and novel object recognition, 2-way ANOVA with Bonferroni post-tests were performed. For H_2_S production capacity assays, 1-way ANOVA with Bonferroni’s multiple comparison test was performed.

## Results

### Late-life EOD fasting on an HFD background reduces total body mass and preserves lean mass compared to AL fed controls

Changes in physical appearance, total body weight, body mass composition in the form of fat and lean masses, and other frailty measurements were observed from baseline to day 76 PDC (Fig. 1a). EOD fasting under the HFD background diminished the oily/greasy appearance of the fur compared to the HFD AL group (Fig. 1b), resulting in a coat of similar appearance as the mice of similar age fed AL and EOD on standard rodent chow diet. Body mass (BM) was gradually and continuously decreased in both the HFD AL and HFD EOD mice during the 2.5 months (Fig. 1c) with HFD AL mice losing approximately 20% and HFD EOD losing approximately 32% (Fig. 1d). Overall, BM loss during the 2.5 months was more pronounced in the HFD EOD group than in the HFD AL group, and followed a similar trend to what was observed between standard chow EOD versus standard chow AL [12] (Supplemental Fig. 1a). While both HFD AL and HFD EOD groups lost weight, it should be noted that changes in BM composition, defined as % of body weight (BW), were not similar. HFD EOD mice maintained greater lean mass (40% BW vs. 38% BW) and lost more fat mass (35% BW vs. 42% BW) compared to HFD AL mice (Fig. 1e). Thus, the EOD fasting regimen in aged mice on the HFD background was sufficient for weight loss by promoting greater fat loss and preserving lean mass compared to HFD AL mice, suggesting the EOD fasting intervention may mitigate the onset and/or severity of aging-related sarcopenia, which is defined as the involuntary loss of skeletal muscle and lean mass [29, 30] and more so present in the HFD AL group.

Consistent with our previous study, we measured the effects of EOD fasting in the HFD group. Food consumption (kcal consumed adjusted to BM) was measured several times over the 78 days of diet intervention to determine average food intake during the fast days (7 total days measured) (Fig. 1f), the fed days (10 total days measured) (Fig. 1g), and combined (7 + 10 = 17 total days measured) (Fig. 1h). As expected, there was a reduction in food intake on the fasted day for the HFD EOD group compared to the HFD AL group. In contrast, on the fed days, the HFD EOD mice had only a slight but not significant increase in food intake compared to HFD AL. Thus, the HFD EOD mice could not completely overcompensate or make up for the missed food intake on the fed days. We also investigated circadian food intake patterns at a higher resolution utilizing metabolic chambers that automatically measured food intake every 20 min over a 4-day span. HFD AL mice consumed food consistently (or at a constant rate) throughout the 24-h period, but predominantly just prior to and during the early dark phase (Supplemental Fig. 1b). While the HFD EOD group obviously consumed no food during the fasting period, they ate the provided food during the 24-h fed period throughout the dark phase as well as a second peak in the second half of the light phase (Supplemental Fig. 1b). Overall, in the course of the 2.5-month intervention, the HFD EOD mice consumed approximately 30% less kcal/food/g body mass compared to HFD AL mice.

### Late-life EOD fasting on HFD background improves carbohydrate utilization and metabolism

**Fig. 2:**
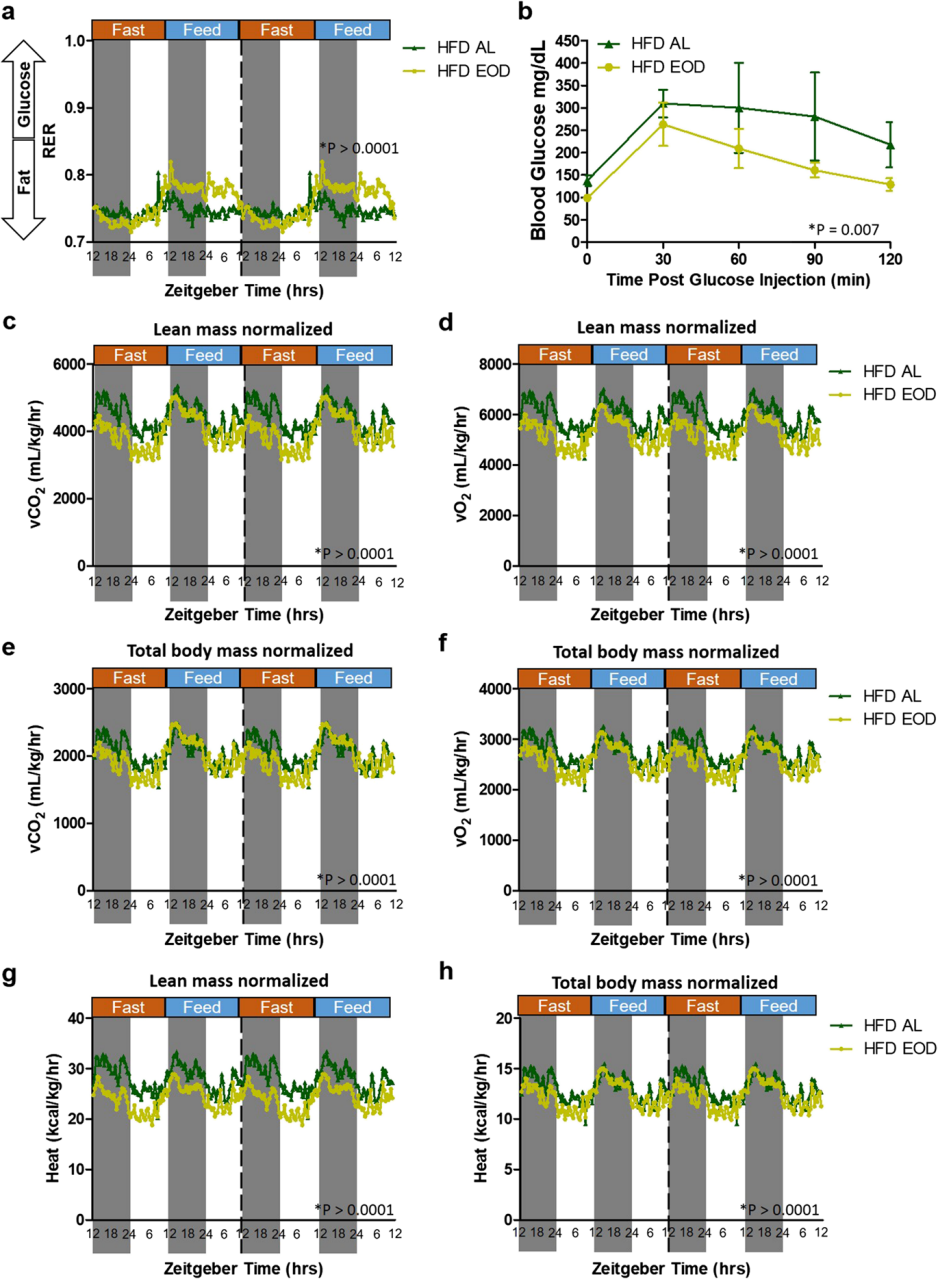
Late-life EOD fasting on HFD background improves carbohydrate utilization and metabolism while lowering overall metabolic output. **a** RER in HFD AL and HFD EOD mice as measured by indirect calorimetry every 20 min over a 4-day period and presented as double-plotted traces of these measurements using the averaged data for each time point within a diet group separated by the fed and fast days with time of the day expressed in ZT and food administration occurring at approximately ZT9 daily and 12:12 light:dark cycle. **b** Average blood glucose levels (mg/dL) at time points between 0 and 120 min following an intraperitoneal injection of glucose from the two GTTs performed on adjacent fasted and fed days in the HFD AL and HFD EOD groups. *N* = 2 mice/HFD AL group and *N* = 3 mice/HFD EOD group, and *p* value analysis was performed as a paired *t*-test between HFD AL versus HFD EOD, with pairing done for each animal’s blood glucose value at each of the 5 time points during the two 120-min GTTs. Data presented as means ± SEM. **c**–**h** The lean mass and total body mass normalized average vCO_2_ (mL/kg/h) (**c**, **e**), vO_2_ (mL/kg/h) (**d**, **f**), and heat (kcal/kg/h) (**g**, **h**) were measured at the same time as the RER in the indirect calorimetry cages as described in **a**. For all metabolic cage data, *N* = 2 mice/HFD AL group and *N* = 3 mice/HFD EOD group, with 220 data points used for each mouse and averaged for each diet group, and tracings depict the mean with no error bars for clarity purpose. The provided *p* values were calculated via paired *t*-tests between HFD AL versus HFD EOD, with pairings between each diet group for each individual animal’s time point reading, with 220 time points/animal in the double-plotted tracing. See also Supplemental Fig. 2

Metabolic activity, flexibility, and glucose utilization were measured using indirect calorimetry. Using this system, we first examined the impact of the EOD fasting on the respiratory exchange ratio (RER), which is calculated by dividing the vCO_2_ produced by the vO_2_ consumed (Fig. 2a). Similar to what we observed in mice under standard chow diet backgrounds [12], the HFD EOD mice had an increased dynamic circadian rhythm of RER by extending the time interval between peaks as well as increased amplitudes of RER during the dark and light phases of the fed days (Fig. 2a). These findings reflect that EOD fasting on the HFD background resulted in a shift from fatty acid to carbohydrate oxidation depending on food availability. These results indicate that EOD fasting initiated late in life even on the HFD background is sufficient to prompt differential fuel utilization and macronutrient oxidation.

Given that HFD EOD fasting amplified the RER closer to 1.0 compared to HFD AL when both had access to food, which is indicative of increased glucose utilization [26], we next tested glucose tolerance handling and clearance using the GTT. Figure 2b shows each time point in the 120-min GTT prior to and post glucose administration compiled and averaged from GTTs performed on adjacent fasted and fed days. Despite there being only a small increase in fasting blood glucose at the 0-min time point for the HFD AL group, the HFD EOD group had enhanced glucose tolerance and clearance compared to the HFD AL groups throughout the 120-min test (Fig. 2b). This improved glucose tolerance was most prominent in the 60- to 120-min post-infusion period. These positive effects of EOD fasting on glucose tolerance were somewhat attenuated when examining the HFD EOD group’s GTT performance data contrasted across fed versus fast day (Supplemental Fig. 2a). Thus, EOD fasting on the HFD background improves glucose utilization and metabolism; however, the results may be somewhat transient and dependent on the GTT being administered after a fed or fasted day.

To determine individual metabolic parameters that go into the RER, such as vO_2_ and vCO_2_, we utilized the OxymaxCLAMS. In doing so, we took into consideration the contributions of the whole body mass as well as just lean mass in impacting these metabolic readouts. This is important, as we show losses in %fat and %lean mass were not equal between the HFD AL and HFD EOD groups (Fig. 1e) and that lean tissue is more metabolically active than fat tissue [31–34]. Thus, we examined our indirect calorimetry data by adjusting vCO_2_, vO_2_, and heat to the individual lean mass or the total body mass of each mouse (Fig. 2c–h). Circadian shifts in vCO_2_, vO_2_, and energy expenditure as measured by heat were observed for both HFD AL and HFD EOD, with elevated outputs detected during the dark phases relative to the light phases. In all readouts regardless of lean mass normalization or total body mass normalization, the HFD EOD group had reduced overall vCO_2_ (Fig. 2c, e), vO_2_ (Fig. 2d, f), and heat production (Fig. 2g, h) throughout all phases, and these were even more prominent during the fast days. Taken together, these findings indicate EOD fasting on the HFD background enhanced glucose utilization and metabolism while also lowering overall metabolic output with decreased vCO_2_, vO_2_, and heat production.

### Late-life EOD fasting on HFD background improves spontaneous activity and neuromuscular coordination

**Fig. 3:**
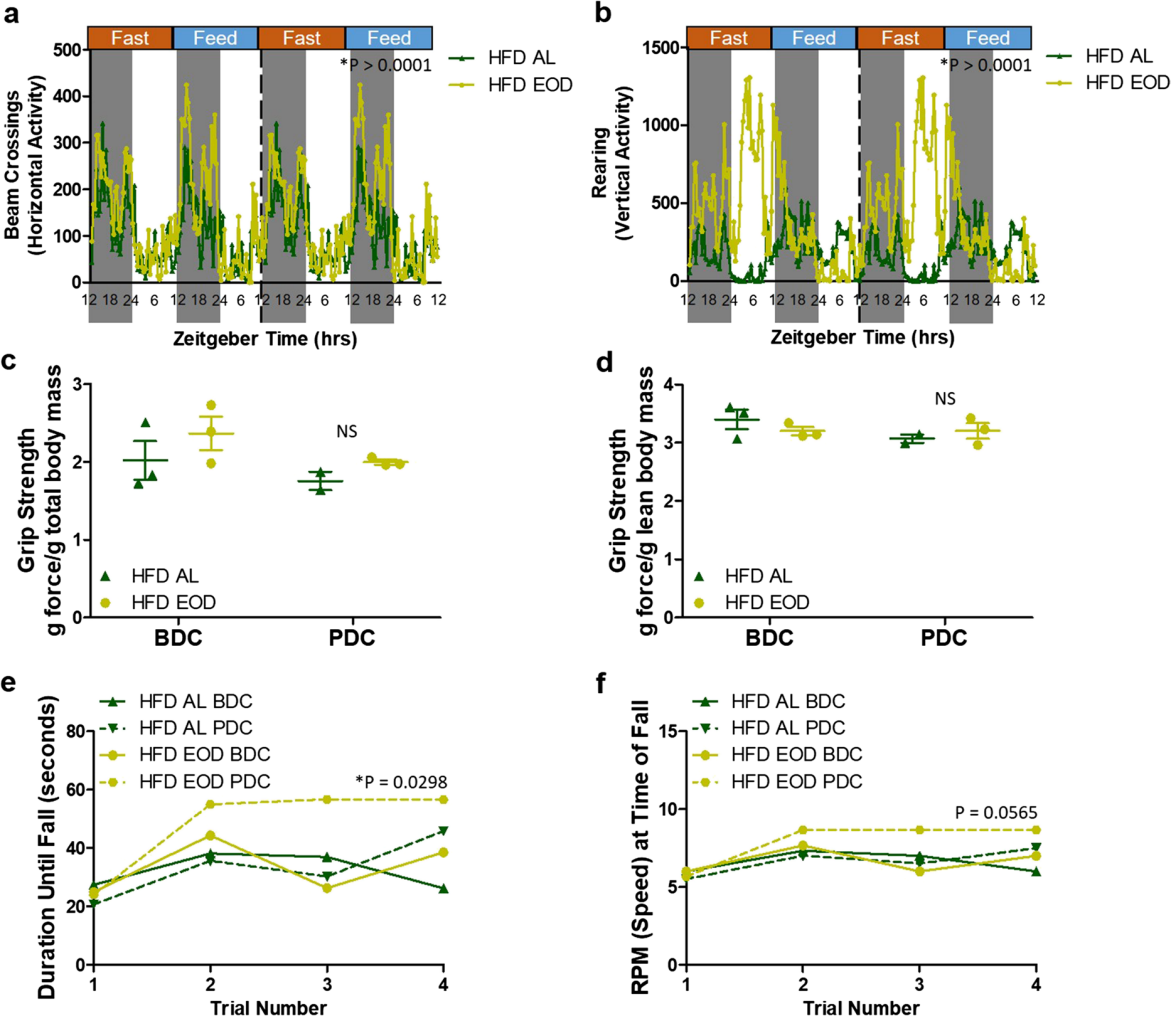
Spontaneous activity, forelimb strength, and neuromuscular coordination in aged mice under HFD AL or HFD EOD fasting diets. **a**, **b** Spontaneous activity as detected in the indirect calorimetry metabolic cages in HFD AL (*N* = 2 mice) and HFD EOD (*N* = 3 mice) by recording beam crossings (horizontal activity; **a**) or rearing (vertical activity; **b**) every 20 min over a 4-day period. Data are presented as double-plotted traces of these measurements using the averaged data for each time point within a diet group separated by the fed and fast days with time of the day expressed in ZT, with a total of 220 data points used for each mouse. The provided *p* values were calculated via paired *t*-tests between HFD AL versus HFD EOD, with pairings between each diet group for each individual animal’s time point data output. **c**, **d** Before diet change (BDC) and post-diet change (PDC) forelimb grip strength adjusted by total body mass (**c**) or lean mass (**d**) in HFD AL and HFD EOD mice. NS, not significant. **e**, **f** BDC and PDC performances in the rotarod test for HFD AL and HFD EOD groups. Plotted are the means of each animal’s duration (**e**) or the speed in RPM (**f**) at time of falling off the rotarod for each of the 4 trials conducted. The provided *p* value refers to the significance of the differences between PDC HFD AL versus PDC HFD EOD and was calculated as a paired *t*-test, with pairings done for each animal’s respective duration and speed values at each of the 4 individual time trials. See also Supplemental Fig. 3

With aging comes decreases in physical activity, strength, and coordination and these are exacerbated under a HFD [35]. Thus, we next examined the impact of late-life initiated EOD fasting under a HFD background on spontaneous activity (Fig. 3a, b), muscular strength (Fig. 3c, d), and neuromuscular coordination (Fig. 3e, f, and Supplemental Fig. 3a). Utilizing again the indirect calorimetry OxymaxCLAMS cages, we monitored spontaneous activity in the form of beam crossings (horizontal movement; Fig. 3a) and rearing (vertical activity; Fig. 3b) in the mice throughout light and dark cycles of both fast and fed periods. In both of these parameters, the HFD EOD group had increased activity compared to HFD AL mice, and this was most noticeable for rearing activity during the light period on fast days demonstrating an increased exploratory behavior (Fig. 3b). Unlike what we previously detected in standard chow EOD mice [12], the HFD EOD mice here did not have total body mass- or lean mass-normalized improvements in the PDC tests for forelimb muscle grip strength (Fig. 3c, d). In the rotarod test, which measures neuromuscular motor coordination, motor learning, and balance [36–39], we detected PDC improvements in duration until fall (Fig. 3e, Supplemental Fig. 3a) and speed at time of fall (Fig. 3f) in the HFD EOD group but not in the HFD AL group. Taken together, these findings suggest that EOD fasting on the HFD background in late life improves spontaneous activity and motor coordination without improving muscular strength.

### Late-life EOD fasting on a HFD background fails to improve hippocampal-dependent memory and behavior along with renal hydrogen sulfide production

**Fig. 4:**
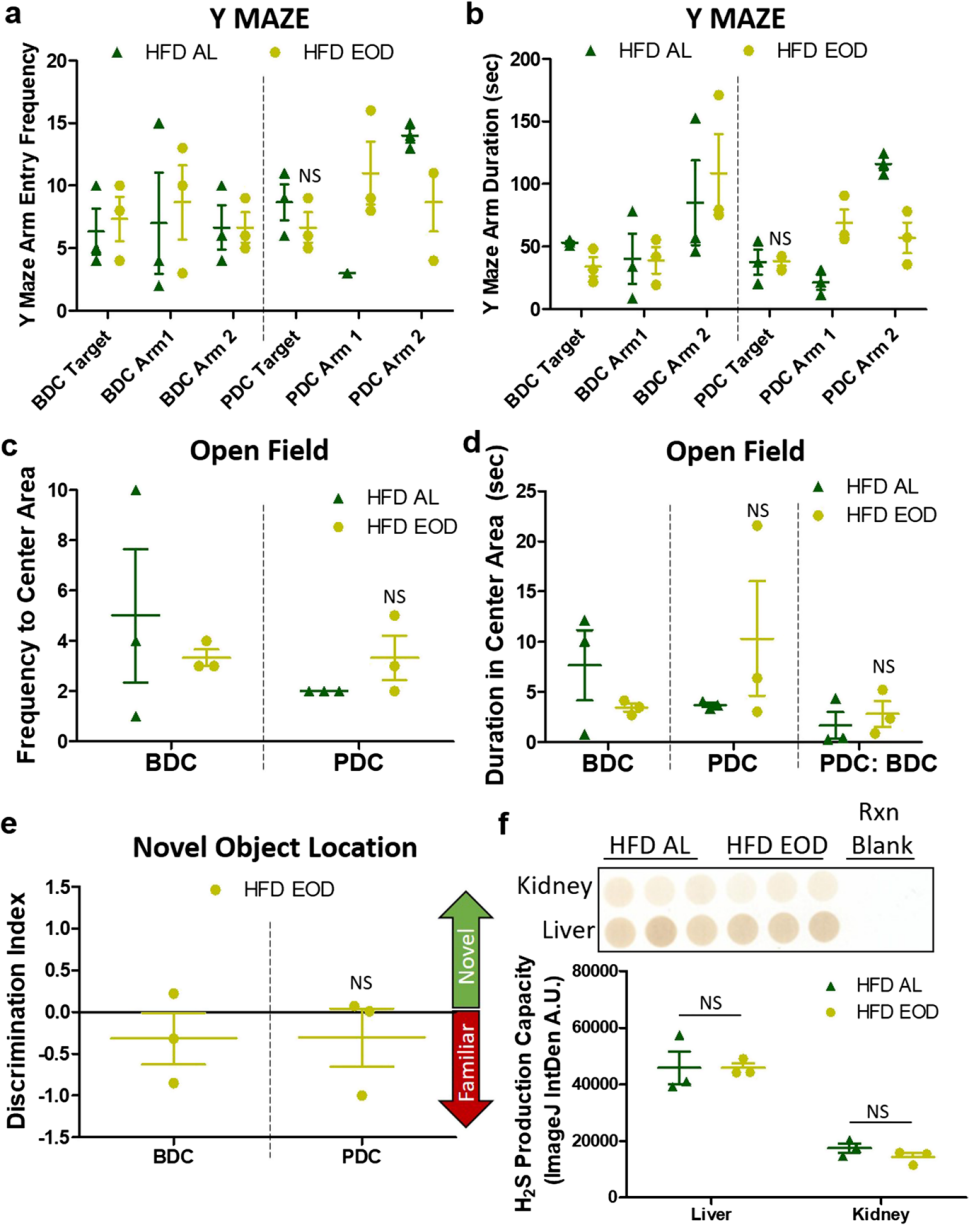
Late-life EOD fasting on a HFD background fails to improve hippocampal-dependent memory and other behavioral endpoints. **a**, **b** Y maze test that examined the number of arm entries (**a**) and the duration of the arm entries (**b**) both at BDC and PDC in the HFD AL and HFD EOD groups. **c**, **d** Open field performance was used to measure anxiety-like behavior BDC and PDC by examining the frequency to center area (**c**) and the duration in the center area (**d**). **e** Long-term object location memory was tested using the novel object location task BDC and PDC. The discrimination index is shown for tests run BDC and PDC in the HFD EOD mice. **f** H_2_S production capacity in the kidney and liver from the PDC HFD AL and HFD EOD groups (*N* = 3) as measured by the filter paper-embedded lead acetate/lead sulfide endpoint assay and quantified with the ImageJ IntDen analysis function with background subtraction from the reaction blank wells. For all plots, *N* = 2–3 mice per group and testing period. The figures depict the mean ± SEM, with NS = not significant. See also Supplemental Fig. 4

As with physical activity capacities, there are declines in learning and memory capacities with age [40, 41]. In our previous study, we determined that late-life initiated EOD fasting under a standard rodent chow diet improved hippocampal-dependent memory in a variety of tests [12]. However, if such an intervention is effective under a HFD background is unknown. Thus, we performed a battery of cognitive/behavior experiments, which included the Y maze, open field, and novel object location tests, to determine if late-life initiated EOD fasting preserves and/or improves hippocampal function and anxiety under an HFD background. First, mice were tested in the Y maze forced alternation task BDC and PDC. In both cases, the HFD AL and HFD EOD mice did not show a preference for the novel target arm over the other two known arms, as there was no increased entry frequency (Fig. 4a) nor duration (Fig. 4b) in the novel target arm. However, there was a numerical but not significant increase in the speed (velocity) the HFD EOD group PDC compared to BDC (Supplemental Fig. 4a).

We next explored the effects of late-life initiated EOD fasting on anxiety-like behavior and locomotion in an open field test BDC and PDC. The frequency of entries (Fig. 4c) and duration (Fig. 4d) to the open field center, along with velocity (Supplemental Fig. 4b) and distance traveled (Supplemental Fig. 4c), were measured. The HFD EOD group did not affect anxiolytic behavior, as there was not an increase in the number of center area entries (Fig. 4c) nor in the duration spent in the center area (Fig. 4d) PDC compared to either BDC or to HFD AL mice. Likewise, the EOD fasting intervention had little impact PDC on the locomotion in the open field test, as there were only minor and not significant increases in speed and distance PDC for the HFD EOD group (Supplemental Fig. 4b, c).

In our final behavioral tasks, we conducted the novel object location (NOL) test BDC and PDC to further examine the impact of HFD EOD fasting on spatial memory. Unlike in the chow EOD mice from our previous experiment [12], the EOD fasting did not affect discrimination index PDC in the HFD group (Fig. 4e), suggesting from our relatively small sample size that a HFD may inhibit the positive effects of EOD fasting on long-term object location memory.

Our previous work related to fasting [12] and environmental endocrine disruption [42] suggested renal hydrogen sulfide (H_2_S) production as a positive factor driving hippocampal-dependent memory and behavior. Notably, we found the aged chow EOD mice from our previous study to have enhanced renal H_2_S production after the dietary intervention [12]. Endogenous H_2_S production and its signaling via protein persulfidation are recognized as potential causal factors in animal models of increased healthspan and lifespan [28, 43–46]. Conversely, there is a loss of H_2_S production and/or sulfide levels in the liver and kidney in rodents [47, 48] and in the circulation of humans [49] as a function of advancing age. Thus, as we failed to detect improvements in hippocampal-dependent memory under the HFD EOD diet, we next probed how aging and the HFD impacted the ability of EOD fasting to modulate tissue-specific H_2_S production. Aging in general caused for a decrease in hepatic H_2_S production capacity in 2-year-old mice compared to 6-month-old mice, and this decrease was made more severe in 2-year-old mice under a HFD (Supplemental Fig. 4d). Likewise, the long-term HFD prevented EOD fasting-induced augmentation of both renal and hepatic H_2_S production capacity (Fig. 4f) which is in contrast to what we previously detected on the lower fat diet chow background [12]. Unexpectedly, we found a slight but significant increase in skeletal muscle H_2_S production capacity from the aged HFD EOD diet mice compared to the HFD AL mice (Supplemental Fig. 4e) that may help explain the improvements in lean mass maintenance, rotarod performance, and spontaneous physical activity detected in this HFD EOD group.

## Discussion

**Fig. 5:**
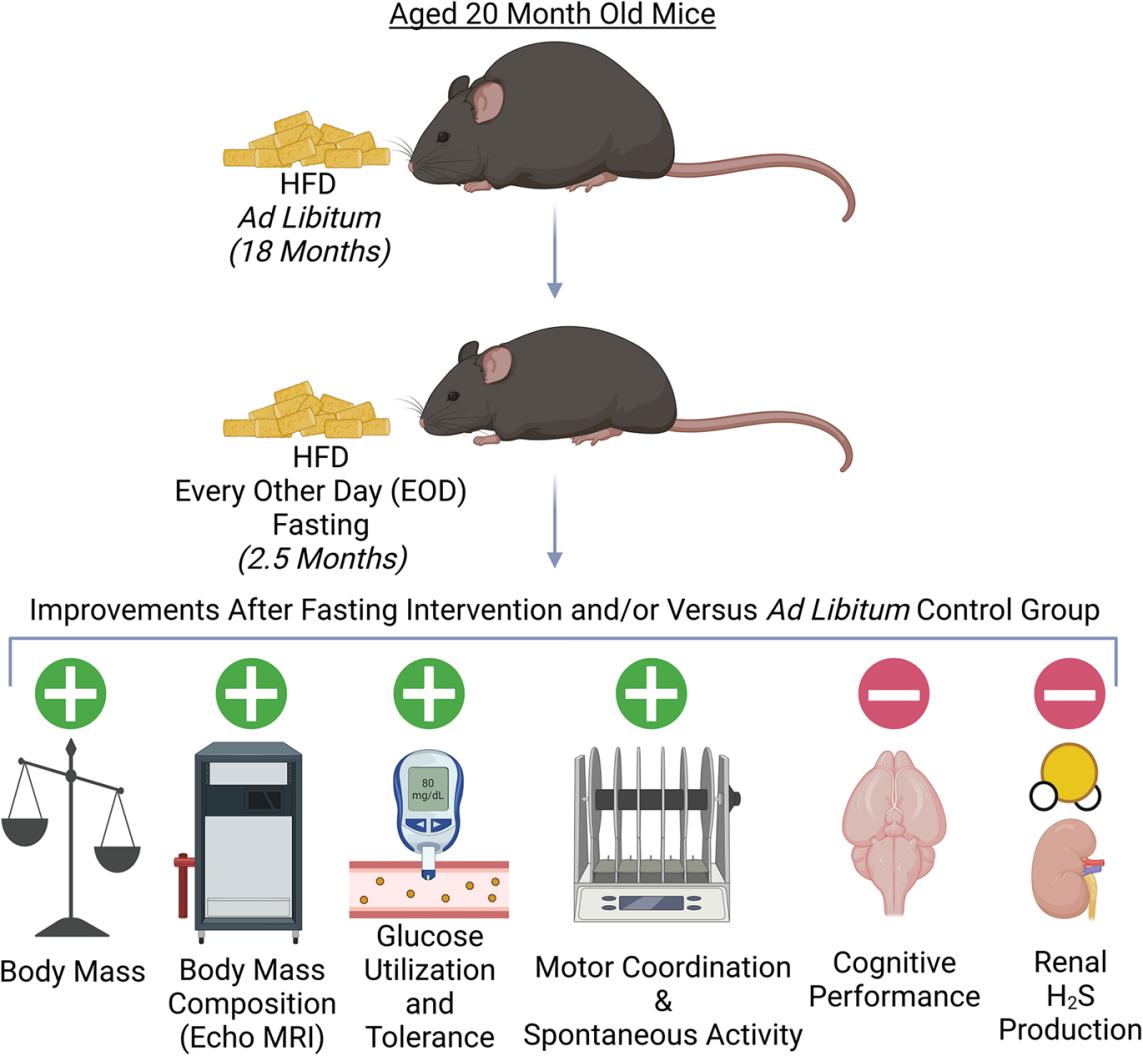
Schematic summary diagram depicting the hypothesized relationship between intermittent fasting and aging-related frailty on a HFD background. Green plus sign indicates improvement, and red minus sign indicates no improvement or no change, after the EOD fasting intervention compared to AL fed controls and/or to pre-intervention status

As a parallel study to our recently published work on late-life intermittent fasting in aged mice on standard composition diet background [12], here we examined if the same 2.5 months of EOD fasting would still be effective in improving multiple frailty measurements on a HFD. Furthermore, unlike our previous study, the mice in the current work at the initiation of the dietary intervention were already obese from 18 prior months of the HFD; thus, we also investigated the ability of the EOD fasting to reverse the physiological, metabolic, and cognitive damages inflicted from the long-term HFD. Consistent with the findings in the standard composition diet background mice [12], the 2.5 months of EOD fasting in obese mice on an HFD diet lowered total body mass, improved lean mass body mass composition relative to fat mass, enhanced glucose utilization and tolerance, and promoted spontaneous physical activity and better motor coordination. In contrast, the EOD fasting on HFD background did not improve muscle strength, hippocampal-dependent memory, or renal H_2_S production. These findings are summarized in Fig. 5.

It has been widely shown for over a century that DR in one form or another without malnutrition stands as one of the best methods to counter both obesity and aging/aging-associated morbidities in animal models and controlled human trials [50–53]. Yet, even with our extensive knowledge on the nutritional, molecular, and temporal mechanisms and triggers for implementing DR for these benefits, it has been incredibly difficult for us as humans to implement these into our everyday lives and adhere to them long term to ultimately reduce energy intake [18, 54]. This hesitancy to adhere to DR is highlighted in recent results from the CALERIE study, in which voluntary subjects aimed for 25% CR over 2 years but ultimately only obtained 14% CR [18, 55, 56]. Despite falling short of the CR goal, the individuals undergoing 14% CR still showed reduced adiposity and improved glucose utilization and insulin sensitivity [18, 55, 56]. Thus, even slight deficits in caloric intake and/or implementation of a meal schedule may provide a defense against obesity. As traditional CR may be difficult to adhere to long term, utilizing other DR regimes that have high compliance, safety, and ease of use will provide a window to address obesity and aging. This is where intermittent fasting, such as the EOD fasting and timed feeding utilized in our previous study [12] and reviewed extensively elsewhere [57–59], presents itself as a somewhat more easily adaptable and viable approach to lower caloric intake and/or constrain feeding periodicity for improving physiological, metabolic, and aging-related endpoints.

While our prior work and the work of others have primarily focused on EOD fasting on a standard non-HFD, there are studies in addition to what is presented here that have examined EOD fasting as a therapeutic approach in clinical trials with obese individuals [60–63] as well as in laboratory rodent models on an HFD background [64–67]. In the human trials on obese female subjects aged 25–65 with a BMI between 30 and 39.9 kg/m^2^ randomized to EOD fasting for 8 weeks on either low-fat diet (LFD) or HFD backgrounds, it was found that EOD fasting was equally as effective in both diet backgrounds for reducing overall weight by ~ 4–5%, diminishing fat mass but not lean mass, and improving coronary heart disease risk factors [60]. These results mirror our results obtained here with HFD EOD fasting as an effective strategy for weight loss and lean mass maintenance. In the rodent tests, it was found that in male C57BL/6 J mice made obese by feeding a 60% HFD for 15 weeks and then undergoing HFD EOD fasting for approximate 2.5 months, the EOD fasting reduced the overall food intake, reduced the overall body mass by approximately 33%, and improved glucose tolerance [66]. These results are in line with those we observed in our current study. However, it should be noted that despite the HFD EOD fasting period of the two studies being similar at 2.5 months in duration, the age of the male mice in the referenced study at initiation of EOD fasting was only 20 weeks of age, while in our study the mice were 20 months of age and on the HFD for the majority of their adult lifetime. Thus, the EOD fasting regimen on an HFD background is effective in both young and old obese mice, as well as humans, for lowering body mass and improving physiological parameters. However, results from EOD fasting on LFD rather than HFD appear to be more beneficial in general and would most likely provide desired outcomes when utilized in humans.

There are several technical and experimental design limitations to our current study. The first includes the small sample sizes of mice in each diet group. This is primarily due to the difficulties of generating large cohorts of mice aged 20 + months that have been on long-term HFD due to accelerated diet-induced mortality. However, the ability to compare the results between AL versus EOD fasting groups at the conclusion of the study on the HFD as well as the BDC and PDC data for the EOD group provides additional robustness and confidence in our findings. Likewise, the parallel study we recently published [12] serves as a thorough comparison to these current findings and emphasizes what physiological, metabolic, and cognitive endpoints are most impacted as a result of dietary composition and EOD fasting in aged mice.

While most endpoints were similarly improved after the 2.5 months of EOD fasting on LFD and HFD, the inability of EOD fasting on the HFD to improve muscle strength and hippocampal-dependent memory is somewhat puzzling and we also do not provide a direct mechanism for these failures. However, as our previous examinations of renal H_2_S production after EOD fasting on a LFD background showed strong positive correlations with behavioral and cognitive performance [12, 42], the lack of such enhancement in renal H_2_S production seen here under the HFD background could provide a potential mechanism for the null cognitive improvement under EOD fasting. These results could indicate that exposure to the long-term HFD irreversibly ablates the ability to improve muscle strength, cognition, and renal H_2_S production at advanced ages, but not for improvements in glucose tolerance, body mass composition, and spontaneous activity. Alternatively, they may require longer dietary intervention than 2.5 months after a lifetime of HFD and it may just be a slowed process for improvements in the former endpoints. Similarly, the use of a more human-relevant 45% kcal HFD [68] versus the 60% kcal HFD used here could possibly shift the results to mirror what was obtained on a LFD and provide better translational appeal for interventions in humans.

Future studies including female cohorts under the HFD background would also be interesting as we previously did not see as great of improvements in frailty endpoints for female mice under EOD fasting as we did for male mice under EOD fasting on the LFD background possibly due to differences in feeding compensation on the fed days and/or hormonal impacts of fasting responses [12]. Nonetheless, the limited number of individual mice, using only males, and using only one strain in this Short Communication presents the need for future studies incorporating larger cohorts of both males and females of diverse strain and genetic backgrounds.

The rising worldwide obesity pandemic, postulated to be caused by a number of genetic, environmental, and lifestyle variables [69–72], necessitates for effective personal, clinical, and public health interventions to counter and reverse the onset of obesity and its associated co-morbidities. Additionally, rapid and continuous declines in physical, metabolic, and cognitive/behavioral health and the advent of frailty from synergisms of obesity and aging require preventative as well as reactionary therapeutic approaches to address both [6–8]. With readily available and easily accessed high-fat/high-calorie foods, dietary interventions to combat both obesity and aging may have to incorporate these types of foods. Thus, the EOD fasting program we have presented here on the HFD background presents as a viable and effective option to counter obesity, even in late life. Future studies examining the effectiveness and safety of EOD fasting in the 6th and 7th decades of life for individuals already suffering from obesity and/or consuming HFDs will need to be further evaluated prior to widespread prescription and adoption of this dietary intervention.

## Supplementary Information

**Additional file1: Supplemental Figure 1.** Every other day (EOD) fasting in aged mice on a HFD background promotes overall weight loss and reduced 48-hour food intake. (a) Absolute body mass in HFD AL (N = 2-3) and HFD EOD (N = 3) mice on Day 0 and Day 76 of the dietary intervention period. Weights from age-matched control chow AL and control chow EOD (N= 5/6/group) were previously determined in [12]; Henderson, et al. *GeroScience* 2021 Aug;43(4):1527-1554. doi: 10.1007/s11357-021-00330-4 and adapted here for comparison to HFD fed mice. Asterisks and provided P value indicate a significant difference between Day 0 and Day 76 for the respective group. Data presented as mean values +/- SEM. (**b**) Food intake in grams as measured in an open-circuit Oxymax Comprehensive Lab Animal Monitoring System (OxymaxCLAMS) every 20 minutes over a 4 day period and presented as double-plotted traces of these measurements using the averaged data for each time point within a diet group separated by the fed and fast days with time of the day expressed in ZT and food administration occurring at approximately ZT9 daily and 12:12 light:dark cycle. N = 2 mice/HFD AL group and N = 3 mice/HFD EOD group, with 220 data points used for each mouse and averaged for each diet group, and tracings depict the mean with no error bars for clarity purpose. Provided P value provided was calculated via paired t-tests between HFD AL versus HFD EOD, with pairings between each diet group for each individual animal’s time point reading. See also companion Figure 1. **Supplemental Figure 2.** Late-life EOD fasting on HFD background improved glucose tolerance is partially dependent on the test being performed on a fed day or fast day. (a) Average blood glucose levels (mg/dL) at time points between 0-120 minutes following an intraperitoneal injection of glucose (2g of glucose/kg of body weight) from the two GTTs performed on adjacent fasted and fed days in the HFD EOD group. Solid trace is from the GTT being performed the morning after the fed day, and the dotted trace is from the GTT being performed the morning after the fast day. In both of these tests, all food was removed and mice placed into clean cages 4-hours prior to the test. N = 3 mice/HFD EOD group, and P value analysis performed as a paired t-test between fed versus fast day, with pairing done for each animal’s blood glucose value at each of the 5 time point during the two 120 minute GTTs. Data points represent the mean value +/- SEM. See also companion Figure 2. **Supplemental Figure 3.** Neuromuscular coordination and learning in aged mice under HFD AL or HFD EOD fasting as determined by the rotarod test. (a) Percent improvement at the Post diet change (PDC) compared to Before diet change (BDC) on an animal to animal average area under the curve (AUC) for time to fall (TTF) from each of the 4 trials conducted at each time point in the rotarod test. NS = not significant. See also companion Figure 3. **Supplemental Figure 4.** Late-life EOD fasting on an HFD background only minimally improves spontaneous movement speed and distance traveled. (a) Spontaneous movement in the Y maze arms, as measured by velocity in mm/sec, at both BDC and PDC in HFD AL and HFD EOD groups. (b-c) In addition to measuring anxiety parameters, the open field test also measured average velocity in mm/sec (b) and total distance traveled as area under the curve (c) for the 10 minutes mice spent in the apparatus BDC and PDC in these Log2 plots. Data is also plotted as a ratio of PDC:BDC in b & c. (d-e) H_2_S production capacity in liver (d) and skeletal muscle (e) (n = 3/group) as measured by the filter paper-embedded lead acetate/lead sulfide endpoint assay and quantified with the ImageJ IntDen analysis function after subtracting the reaction mix blank wells. (d) H_2_S production in livers from young 6 month mice on AL low fat chow diet versus old 2 year mice on AL low fat chow diets or HFD and associated quantification and statistical analysis. (e) H_2_S production from skeletal muscle (quadriceps) from PDC HFD AL and HFD EOD groups. For all plots, N = 2-3 mice per group and testing period. The figures depict the mean ± SEM, with NS = not significant. See also companion Figure 4.

## Data Availability

Data supporting the reported results and conclusions can be found in the main figures and supporting supplemental figures and data files. Requests for additional research materials will be fulfilled from the corresponding author (CH): hinec@ccf.org.
