# Supplementary material for: A long-term obesogenic high-fat diet in mice partially dampens the anti-frailty benefits of late-life intermittent fasting
Source: GeroScience. 2022 Oct 26;45(2):1247–62. doi: 10.1007/s11357-022-00678-1 (PMC9886776; doi:10.1007/s11357-022-00678-1)
Supplement: Supplementary file 1 — Additional file1: Supplemental Figure 1. Every other day (EOD) fasting in aged mice on a HFD background promotes overall weight loss and reduced 48-hour food intake. (a) Absolute body mass in HFD AL (N = 2-3) and HFD EOD (N = 3) mice on Day 0 and Day 76 of the dietary intervention period. Weights from age-matched control chow AL and control chow EOD (N= 5/6/group) were previously determined in [12]; Henderson, et al. GeroScience 2021 Aug;43(4):1527-1554. doi: 10.1007/s11357-021-00330-4 and adapted here for comparison to HFD fed mice. Asterisks and provided P value indicate a significant difference between Day 0 and Day 76 for the respective group. Data presented as mean values +/- SEM. (b) Food intake in grams as measured in an open-circuit Oxymax Comprehensive Lab Animal Monitoring System (OxymaxCLAMS) every 20 minutes over a 4 day period and presented as double-plotted traces of these measurements using the averaged data for each time point within a diet group separated by the fed and fast days with time of the day expressed in ZT and food administration occurring at approximately ZT9 daily and 12:12 light:dark cycle. N = 2 mice/HFD AL group and N = 3 mice/HFD EOD group, with 220 data points used for each mouse and averaged for each diet group, and tracings depict the mean with no error bars for clarity purpose. Provided P value provided was calculated via paired t-tests between HFD AL versus HFD EOD, with pairings between each diet group for each individual animal’s time point reading. See also companion Figure 1. Supplemental Figure 2. Late-life EOD fasting on HFD background improved glucose tolerance is partially dependent on the test being performed on a fed day or fast day. (a) Average blood glucose levels (mg/dL) at time points between 0-120 minutes following an intraperitoneal injection of glucose (2g of glucose/kg of body weight) from the two GTTs performed on adjacent fasted and fed days in the HFD EOD group. Solid trace is from the GTT being performed th [file 11357_2022_678_MOESM1_ESM.docx]

Supplemental Figures and Legends for “**A Long-term Obesogenic High Fat Diet in Mice Partially Dampens the Anti-Frailty Benefits of Late-Life Intermittent Fasting**” by Henderson, *et al.*

**Supplemental Figure 1.**

**
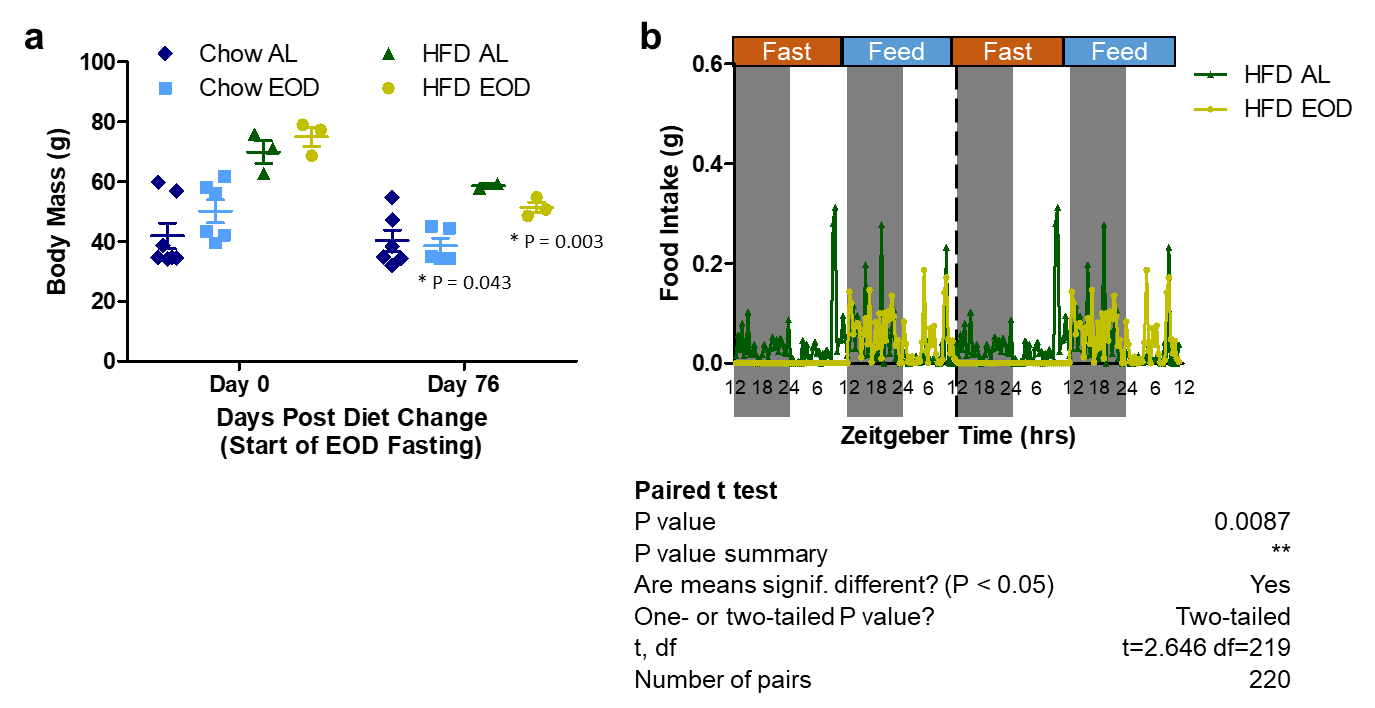
**

**Supplemental Figure 1. Every other day (EOD) fasting in aged mice on a HFD background promotes overall weight loss and reduced 48-hour food intake. (a)** Absolute body mass in HFD AL (N = 2-3) and HFD EOD (N = 3) mice on Day 0 and Day 76 of the dietary intervention period. Weights from age-matched control chow AL and control chow EOD (N= 5/6/group) were previously determined in [12]; Henderson, et al. *GeroScience* 2021 Aug;43(4):1527-1554. doi: 10.1007/s11357-021-00330-4 and adapted here for comparison to HFD fed mice. Asterisks and provided P value indicate a significant difference between Day 0 and Day 76 for the respective group. Data presented as mean values +/- SEM. (**b**) Food intake in grams as measured in an open-circuit Oxymax Comprehensive Lab Animal Monitoring System (OxymaxCLAMS) every 20 minutes over a 4 day period and presented as double-plotted traces of these measurements using the averaged data for each time point within a diet group separated by the fed and fast days with time of the day expressed in ZT and food administration occurring at approximately ZT9 daily and 12:12 light:dark cycle. N = 2 mice/HFD AL group and N = 3 mice/HFD EOD group, with 220 data points used for each mouse and averaged for each diet group, and tracings depict the mean with no error bars for clarity purpose. Provided P value provided was calculated via paired t-tests between HFD AL versus HFD EOD, with pairings between each diet group for each individual animal’s time point reading. See also companion Figure 1.

**Supplemental Figure 2.**


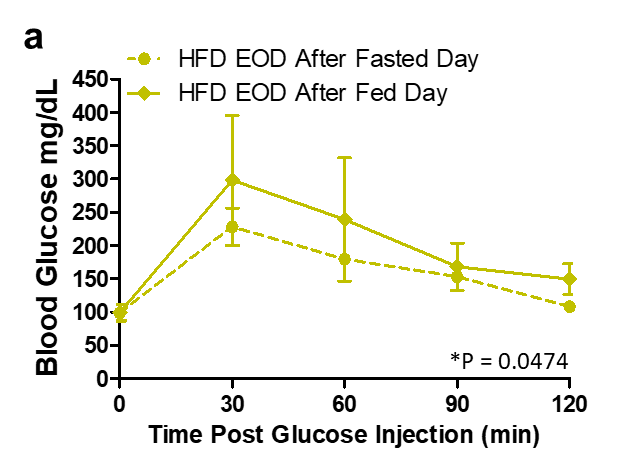


**Supplemental Figure 2. Late-life EOD fasting on HFD background improved glucose tolerance is partially dependent on the test being performed on a fed day or fast day. (a)** Average blood glucose levels (mg/dL) at time points between 0-120 minutes following an intraperitoneal injection of glucose (2g of glucose/kg of body weight) from the two GTTs performed on adjacent fasted and fed days in the HFD EOD group. Solid trace is from the GTT being performed the morning after the fed day, and the dotted trace is from the GTT being performed the morning after the fast day. In both of these tests, all food was removed and mice placed into clean cages 4-hours prior to the test. N = 3 mice/HFD EOD group, and P value analysis performed as a paired t-test between fed versus fast day, with pairing done for each animal’s blood glucose value at each of the 5 time point during the two 120 minute GTTs. Data points represent the mean value +/- SEM. See also companion Figure 2.

**Supplemental Figure 3.**


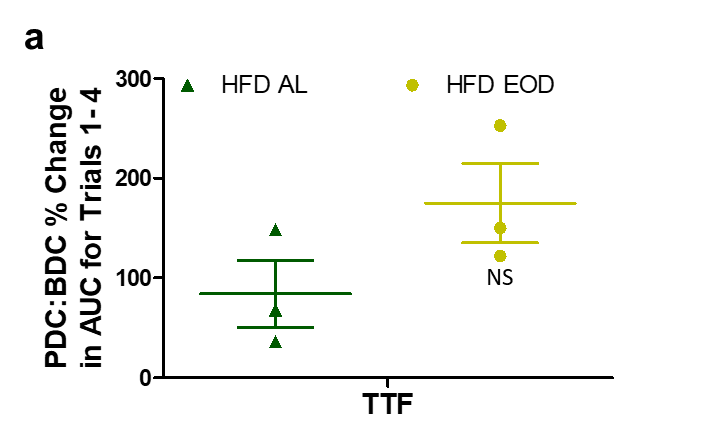


**Supplemental Figure 3: Neuromuscular coordination and learning in aged mice under HFD AL or HFD EOD fasting as determined by the rotarod test. (a**) Percent improvement at the Post diet change (PDC) compared to Before diet change (BDC) on an animal to animal average area under the curve (AUC) for time to fall (TTF) from each of the 4 trials conducted at each time point in the rotarod test. NS = not significant. See also companion Figure 3.

**Supplemental Figure 4.**


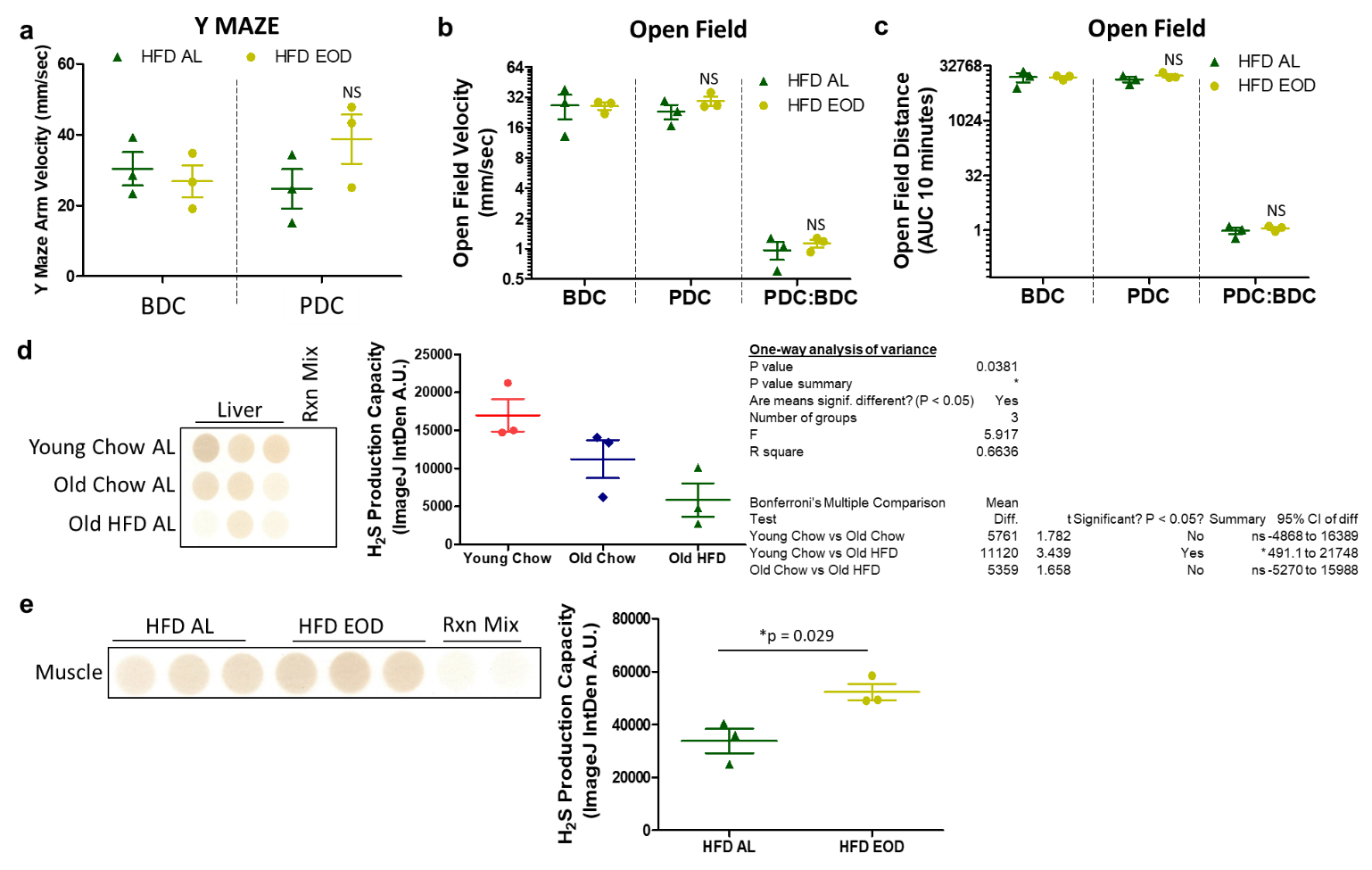


**Supplemental Figure 4. Late-life EOD fasting on an HFD background only minimally improves spontaneous movement speed and distance traveled. (a)** Spontaneous movement in the Y maze arms, as measured by velocity in mm/sec, at both BDC and PDC in HFD AL and HFD EOD groups. (**b-c**) In addition to measuring anxiety parameters, the open field test also measured average velocity in mm/sec (**b**) and total distance traveled as area under the curve (**c**) for the 10 minutes mice spent in the apparatus BDC and PDC in these Log2 plots. Data is also plotted as a ratio of PDC:BDC in b & c. **(d-e)** H_2_S production capacity in liver (**d**) and skeletal muscle (**e**) (n = 3/group) as measured by the filter paper-embedded lead acetate/lead sulfide endpoint assay and quantified with the ImageJ IntDen analysis function after subtracting the reaction mix blank wells. (**d**) H_2_S production in livers from young 6 month mice on AL low fat chow diet versus old 2 year mice on AL low fat chow diets or HFD and associated quantification and statistical analysis. (**e**) H_2_S production from skeletal muscle (quadriceps) from PDC HFD AL and HFD EOD groups. For all plots, N = 2-3 mice per group and testing period. The figures depict the mean ± SEM, with NS = not significant. See also companion Figure 4.
